# Letter from Paris

**Published:** 1868-06

**Authors:** D'Oyley Evans


### ARTICLE VII.

### *Letter from Paris.*

2 PLACE DE LA TRINITE,

PARIS, *March* 11, 1868.

DEAR DOCTEUR :—

I lately perused a letter from Percy Boulton M. D., of London upon the after-treatment of scarlet fever and as that maladie is rather prevalent among American children I will send you an exact copy of Dr. B's letter, which if you see fit you can insert in the next number of the American Journal of Dental Science.

"The sequelae of scarlet fever are so frequently the dread of both the medical man and the mothers of the little ones, who suffer from the disease,that I venture to lay before the profession a line of treatment which in my hands has been followed with wonderful success. I do not doubt that others have used the same or similar remedies, but so far as I am aware, the profession generally is not sufficiently impressed with the almost specific nature of the treatment which I advocate, and as there has been, and still is, a considerable amount of scarlet fever about at this season of the year, I think the present a favorable time for making known my views.

After an attack of the scarlet fever the little patient is usually left in a cachetic condition with enlargement of the cervical glands, and an impoverished state of the blood, which too often is followed by some strumous affection, by mucous discharges from one or other of the passages, by nephritis, dropsy, rheumatism, or pericarditis. Now when we consider the complications which may arise, and then remember the property of the drug which I use, the wonder is that it is not the universal treatment pursued by the profession.

"Iodine," say Squire "acts especially as a stimulant to the entire lymphatic system, causing absorption, promoting elimination by the kidneys; acting as an antidote to certain blood poisons, organic and inorganic; also in chronic inflammation, promotes absorption and elimination in dropsies and chronic rheumatism; most efficacious in glandular enlargements, scrofulous glands of the neck, or as an alternative in obstinate mucous discharges." During the convalescence of all cases of scarlet fever, I use iodine either in the form of a syrup of the iodide of iron, or as iodide of potassium. I find as a rule, the former preferable, because children like it on account of its sweetness, and I think the iron a good and useful combination. I have never seen anything but the best results, caution as to clothing and the time allowed to go into the open air being enjoined. I sometimes use iodine to a very much enlarged plan externally. I think, with one exception, I have never seen a gland suppurate, and this case did not come under my care till too late. I have seen children who were reduced to a skeleton rally in a miraculous way after the use of the iodide; immensely enlarged cervical glands disappearing, and rosy cheeks and sparkling eyes taking the place of the ghostly remains of the former little one.

Every day the antiseptic treatment of disease is coming more into favor, and the day may not be far distant when we shall be able to destroy all poisons in their early stages, and thus free ourselves of the whole class of zymotic diseases.

In the meantime however, we are glad to know of anything which will in any way lessen the danger of so virulent a poison, and I recommend the use of Iodine as prescribed by myself after all cases of scarlet fever, with the greatest confidence." I have thought the above letter worthy of careful attention and shall be delighted if a drug which I have so thoroughly tested for diseases of the mouth, and have been so successful with it in my practice, will also prove as efficacious in the after treatment of that distressing malady which is the terror of parents and is so prevalent in my native land.

Dr. Percy Boulton gives his information with such unboundeb confidence, that I hope some eminent practitioner in America will be as successful with the same application and thus reduce the many strumous troubles that become so injurious to the general health of persons that have suffered from scarlet fever in their childhood.

In my next letter I will send on some notes relating to a new application of Phenic or Carbolic acid, for small-pox.

If perfectly successful it will be an invaluable remedy, and from all that I have learnt I have great confiidence in its efficacy.

Ever yours respectfully

D'Oyley Evans.

---

